# Chronological changes of viral shedding in adult inpatients with Omicron infection in Shanghai, China

**DOI:** 10.3389/fimmu.2023.1090498

**Published:** 2023-02-01

**Authors:** Xinru Zhou, Xiaochun Huang, Tingting Sun, Xiaolan Jin, Zhaofeng Tian, Miao Xue, Jinsong Kang, Bai Gao, Aijing Xu, Yi Chen, Yin Jia, Shanrong Liu

**Affiliations:** ^1^ Department of Laboratory Diagnostics, Changhai Hospital, Second Military Medical University, Shanghai, China; ^2^ Information Department, Changhai Hospital, Second Military Medical University, Shanghai, China; ^3^ Infectious Department, Changhai Hospital, Second Military Medical University, Shanghai, China

**Keywords:** coronavirus disease 2019, severe acute respiratory syndrome coronavirus 2, Omicron, viral load, quantification cycle

## Abstract

**Background:**

Coronavirus disease 2019 (COVID-19) caused by the Omicron variant occurred in Shanghai, China, but its clinical characteristics and virology have not been comprehensively described.

**Methods:**

This retrospective cohort study included adult inpatients (≥18 years) diagnosed with COVID-19 at Changhai Hospital. Laboratory and clinical data were obtained from electronic medical records to investigate the clinical characteristics of COVID-19 and the variations in the patients’ laboratory indexes were examined.

**Results:**

The symptoms of COVID-19 caused by the Omicron variant were relatively mild. Upper respiratory tract specimens yielded higher positive detection rates than lower respiratory tract and intestinal specimens. Peak COVID-19 viral load was reached at the time of admission; quantification cycle (Cq) values increased to approximately 35 after 8.54 days. *In vivo* viral shedding duration correlated with age and disease severity (*p*<0.05). The older the patient and the more severe the disease, the longer the duration of viral shedding was. Portion parameters of blood routine, coagulative function, clinical chemistry, and inflammatory factor showed a certain correlation with the SARS-CoV-2 viral load.

**Conclusions:**

Virus replication and shedding are rapid in Omicron-positive patients; COVID-19 in these patients is characterized by acute onset, mild symptoms, and fast recovery. Older patients and those with more severe disease demonstrate prolonged virus shedding. Routine hematological indexes can reveal disease severity and help clinically evaluate the patient’s condition.

## 1 Introduction

Severe acute respiratory syndrome coronavirus 2 (SARS-CoV-2), which causes coronavirus disease 2019 (COVID-19), has rapidly spread worldwide, becoming a global pandemic (1, 2). As SARS-CoV-2 spread among humans continues, new variants with different transmissibility, pathogenicity, and antibody evasion capacities continuously emerge. Currently, the World Health Organization has confirmed five SARS-CoV-2 variants of concern (VOC), namely, Alpha (B.1.1.7), Beta (B.1.351), Gamma (P.1), Delta (B.1.617.2), and Omicron (B.1.1.529) (3). An outbreak of the Omicron variant has been ongoing in Shanghai, China, since March 2022, and sequencing studies have confirmed the involvement of Omicron variants BA.2 and BA.2.2 (4). In addition, two samples from this study were blindly selected for macrogenomic testing, and bioinformatics analysis suggested that the Omicron variant in both specimens was the BA.2.2.1 subtype (**Supplemental Figure S1**). Previously, researchers have described other variants’ clinical characteristics and virological processes (5–7); however, details about the circulating Omicron variant’s epidemiological characteristics and virological processes have not been comprehensively released.

Nucleic acid testing is critical for controlling epidemics and confirming SARS-CoV-2 infections. The reverse transcription quantitative real-time polymerase chain reaction (RT-qPCR) analysis is widely used to measure the Cq, and this semi-quantitative method is used to confirm SARS-CoV-2 infection. SARS-CoV-2 virus shedding kinetics is important for determining whether the patient has become negative and for clinical diagnosis and treatment. Furthermore, routine hematological indexes are correlated with disease severity and can help determine viral infection severity. This study investigated the clinical characteristics of hospitalized patients with COVID-19, *in vivo* viral load variation trends, and the relationship between routine hematological indexes and changes in patient conditions. The results of the study should provide a laboratory basis for the diagnosis, treatment, and monitoring of COVID-19 patients.

## 2 Materials and methods

### 2.1 Study subjects and data collection

This was a retrospective cohort study. The study data were mainly obtained from the electronic case system database at Changhai Hospital and comprised the medical statistical data regarding all inpatients. The experimental data were masked to ensure patient privacy. All data were independently checked by two study staff for accuracy. This study was approved by the medical ethics committee of Changhai Hospital (approval no. CHEC2022-067).

### 2.2 Study design and subject statistical data

The ID numbers on the first page of the inpatient notes extracted from the database were used as unique patient identifiers. The discharge diagnosis was used to group subjects into the mild, general, and severe COVID-19 groups. The diagnostic criteria were based on the COVID-19 diagnosis and treatment protocol (interim 9th edition) (8). Mild COVID-19 was defined as mild clinical symptoms and pneumonia absent on radiologic examination. General COVID-19 was defined as mild clinical symptoms with pneumonia present on radiologic examination. Severe COVID-19 was diagnosed when adult patients met any of the following criteria: 1) shortness of breath, RR ≥30 breaths/minute, 2) oxygen saturation **≤**93% during inhalation at rest, 3) arterial blood pressure (PaO_2_)/fraction of inspired oxygen (FiO_2_) ≤300 mmHg (1 mmHg=0.133 kPa) (PaO_2_/FiO_2_ was corrected using the following formula for high altitude (altitude **>**1000 m) regions: PaO_2_/FiO_2_ ×[760/atmospheric pressure (mmHg)]), and 4) progressive worsening of clinical symptoms with lung radiology showing an **>**50% significant progression in the lesion within 24–48 hours (8). The discharge criteria were as follows: 1) normal temperature for 3 days or more, 2) a significant improvement in respiratory symptoms, 3) lung imaging showing significant improvement in acute exudative lesions, and 4) two consecutive SARS-CoV-2 nucleic acid tests for the ORF1ab gene (hereinafter referred to as the O gene) and N gene with a Cq ≥35 (RT-qPCR) and a sampling interval of at least 24 hours. Patients who met the above criteria could be discharged (8). The exclusion criterion used in this study was age **<**18 years.

### 2.3 Sampling and test method

There were three types of nucleic acid test specimens, namely, nasopharyngeal and oropharyngeal swab, induced sputum, and anal swab. After sampling, the specimen was placed in a disposable virus sampling tube (containing 3 ml of virus preservation solution, Shandong Yida). The COVID-19 diagnosis and treatment protocol (interim 9th edition) was used for nasopharyngeal and oropharyngeal swabs and anal swabs. The sampling method for induced sputum was as follows: 1) 5 mg/2.5 ml salbutamol sulfate nebulization solution was added to a disposable nebulizer and inhaled for 3–5 minutes; 2) the patient was instructed to rest for 3 minutes before 2 vials of 0.9% sodium chloride were added to 1 vial of 10% sodium chloride and mixed evenly; subsequently, 2 ml of the solution was added to a disposable nebulizer, nebulization was continued, and 3) the patient was instructed to cough sputum into a disposable sputum cup, from which 1 ml sputum was removed and added to the virus sampling tube. RT-qPCR (9, 10) was used to measure nucleic acid levels in all specimens. The hospital laboratory staff performed standardized testing according to the assay kit and device manufacturers**’** directions. The nucleic acid extraction reagent and amplification reagent were manufactured by Shanghai ZJ Biotechnology (China). The model number of the fully automated nucleic acid extractor was EX9600 (Shanghai ZJ Bio-Tech). The RT-qPCR machine used was LightCycler 480 II (Roche, Switzerland). Positive nucleic acid test results were presented as Cq values (inversely proportional to viral load). The determination criteria for positive results were as follows: Cq **≤**42 for either the O or N gene. In this study, negative results were shown as Cq =43 to facilitate statistical analyses. Samples were tested for routine hematological indexes according to the standard operating procedure of the laboratory. Some laboratory test results were in the form of numerical intervals, and such a result was replaced with the mid-value of the interval to facilitate statistical analysis. For example, CRP <0.5 mg/L was replaced by CRP =0.25 mg/L.

### 2.4 Statistical analysis

Continuous variables are expressed as the median and interquartile range (IQR). Categorical variables are expressed as count and percentage. A two-tailed difference of *p*<0.05 was deemed to indicate statistical significance. T-tests and ANOVA were used for parametric data analyses. The Kruskal–Wallis test was used for non-parametric data. GraphPad Prism (version 8.0) was used for all analyses. Microsoft Excel (2206 version) was used to generate polynomial fitted curves with R^2^ as the evaluation criterion.

## 3 Results

### 3.1 Patient characteristics

The case data and test results of 176 COVID-19 patients hospitalized from April 1 to May 24, 2022, were collected. Every patient underwent at least five nasopharyngeal and oropharyngeal swab nucleic acid tests; the total number of specimens was 2700. The median patient age was 33 years (19–95 years). Sixty-six percent of patients were younger than 40 years. Male patients accounted for 57%. Seventy-seven percent of the patients had clinical symptoms. The most common symptoms were cough/dry cough (43%), followed by dry throat/sore throat (36%) and fever (35%). There were 154 (87%), 17 (10%), and 5 (3%, all **>**85 years of age) patients with mild, general, and severe COVID-19, respectively. In addition, 26 (15%), 7 (4%), 5 (3%), and 4 (2%) patients had comorbid hypertension, coronary heart disease, diabetes, and autoimmune disease, respectively. One hundred sixty-one patients (91%) had an interval of **≤**4 days between the onset of symptoms to registration and admission. The turnover period of most of these patients was 1–2 days. For treatment, traditional Chinese medicines were used for 94% of the patients, including Xuanfei Zhisou decoction, Lianhua Qingwen granules, and Shufeng Jiedu capsules, of which Xuanfei Zhisou decoction was used most frequently (68% of the patients). Two patients with comorbid COPD had long disease duration and slow viral shedding (**Supplemental Figure S2**). In addition, 67 (38%) of patients received the antiviral drug molnupiravir. However, our results showed no significant difference in the course of disease between patients taking and not taking molnupiravir (only 5 patients with severe disease were not included) (**Supplemental Figure S3**). Respiratory support was necessary for 3 patients with severe COVID-19. **Table 1** summarizes the characteristics of the above patients.

**Table 1:** Patient characteristics.

Characteristics	Data (*N*=176)
Age, median (range), years	33 (19–95)
<40, *n* (%)	117 (66)
[40, 60], *n* (%)	31 (18)
>60, *n* (%)	28 (16)
Sex, male/female, n	101/75
Symptom, n (%)	135 (77)
Fever	62 (35)
Cough/dry cough	76 (43)
Productive cough	26 (15)
Headache/dizziness	16 (9)
Nasal congestion/rhinorrhea	11 (6)
Dry throat/sore throat	63 (36)
Fatigue/drowsiness	11 (6)
Muscle soreness	16 (9)
Hyposmia/hypogeusia	5 (3)
Chills	3 (2)
Diarrhea	1 (1)
Chest tightness	2 (1)
Disease severity, n (%)	
Mild	154 (87)
General	17 (10)
Severe	5 (3)
Critical	0 (0)
Complications, n (%)	37 (21)
Hypertension	26 (15)
Diabetes	6 (3)
Coronary heart disease	7 (4)
Chronic obstructive pulmonary disease	3 (2)
Tumor	0 (0)
Autoimmune disease ^a^	4 (2)
Interval from disease onset to registration and admission, median (range), days	1 (1–14)
≤4, *n* (%)	161 (91)
>4, *n* (%)	15 (9)
Treatment, n (%)	
Antiviral treatment^b^	67 (38)
Intravenous infusion of immunoglobulin	2 (1)
Immunotherapy	2 (1)
Respiratory support^c^	3 (2)
Renal replacement therapy	1 (1)
Traditional Chinese medicine, n (%)	166 (94)
Lianhua Qingwen granules	96 (55)
Xuanfei Zhisou decoction	119 (68)
Shufeng Jiedu capsules	62 (35)
Riye Baifuning	82 (47)
Clinical outcome at data collection cutoff	
Discharge	176 (100)
Death	0 (0)
Hospitalized	0 (0)

^a^Four patients had comorbid autoimmune disease, one had comorbid rheumatoid arthritis (RA), one had comorbid connective tissue disease (CTD), and two had comorbid chronic obstructive pulmonary disease (COPD).

^b^The antiviral drug was molnupiravir.

^c^Three patients received transnasal high-flow oxygen. After that, one patient was switched to endotracheal intubation and assisted ventilation, and another patient was switched to low-flow oxygen.

### 3.2 Comparison of viral load among different types of specimens

**Figure 1:**
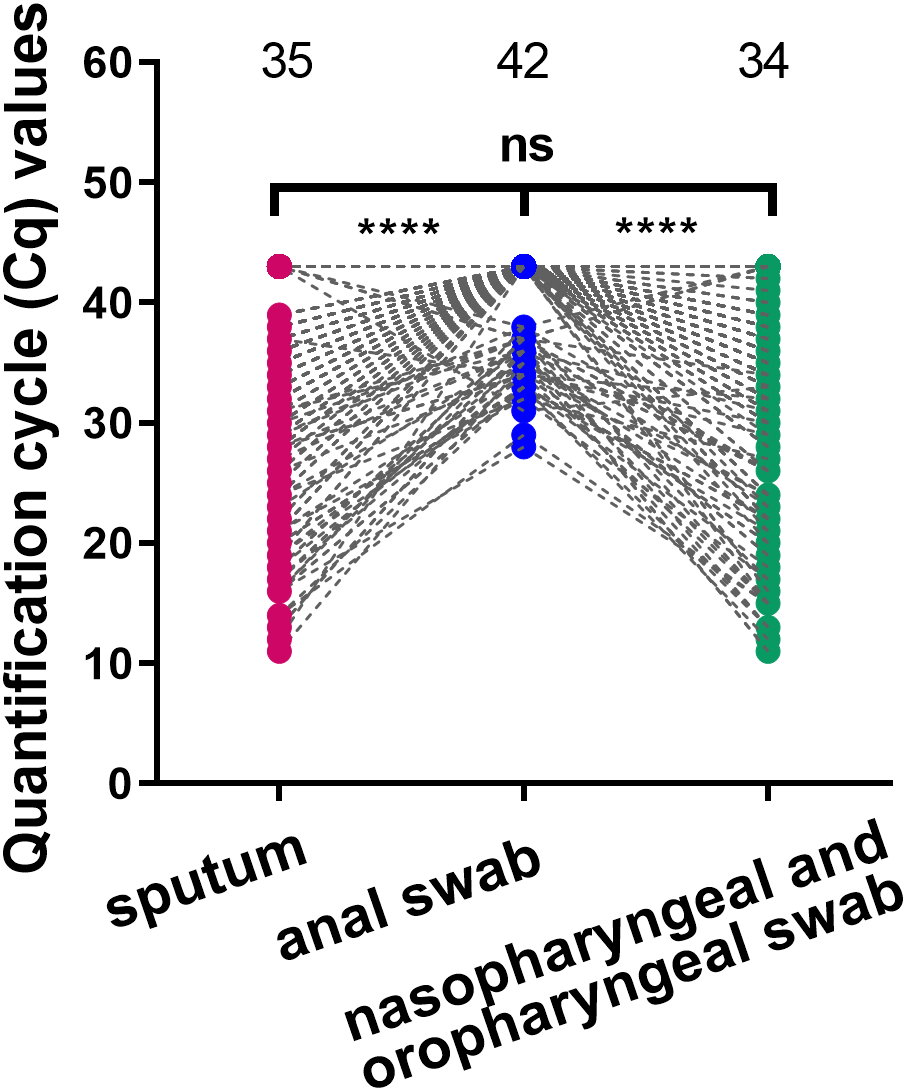
Comparison of viral load in different types of specimens. Comparison of viral load among nasopharyngeal and oropharyngeal swab, induced sputum and anal swab specimens collected at the same time point. ****p<0.0001, ns: not significant. The three numbers at the top of the picture correspond to the mean of Cq value of each group.

To confirm that the best sample was used for SARS-CoV-2 testing, we compared the viral load of three types of specimens collected from the same patient during the same period. There were 360 groups and 1080 specimens in total. As shown in **Supplementary Table S1**, the positive detection rate was the highest for nasopharyngeal and oropharyngeal swabs (78%, 282/360), followed by the induced sputum specimen (69%, 248/360); the positive detection rates of these two specimens were significantly higher than those obtained using anal swabs (11%, 41/360). Paired t-test results showed that the mean Cq value of the anal swab was higher than that of nasopharyngeal and oropharyngeal swabs or induced sputum samples (*p*<0.0001) (**Figure 1**), showing that viral load was the lowest in the anal swab. There was no statistically significant difference between the mean Cq values of nasopharyngeal and oropharyngeal swab and induced sputum samples (*p*=0.2205) (**Figure 1**). The above results showed that the positive detection rate and viral load of respiratory tract samples were higher than those of intestinal samples, and the positive detection rate of upper respiratory tract samples was higher than that of lower respiratory tract samples.

### 3.3 General virus shedding trends after admission

**Figure 2:**
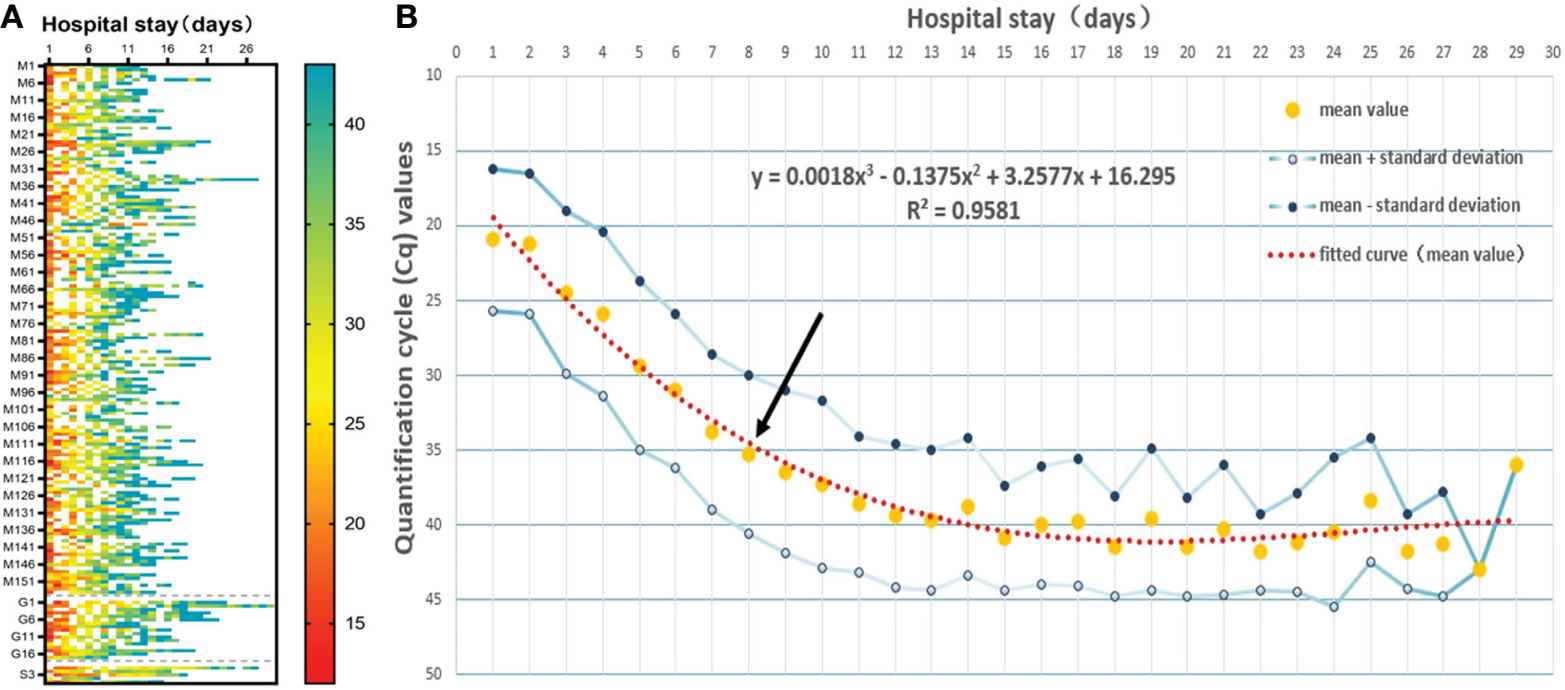
Viral clearance trends over time after admission. **(A)** The heat map showed the Cq values measured in all study subjects after admission. M indicates mild disease, G indicates general disease, and S indicates severe disease. **(B)** The mean Cq values of all study subjects at different time points after admission were used for the polynomial fitted curve analysis to reveal the viral clearance variation trends over time. Black arrow shows the intersection between the polynomial fitted curve and the Y-axis at 35.

Since nasopharyngeal and oropharyngeal swabs had the higher positive detection rate, we selected Cq values from nasopharyngeal and oropharyngeal swab specimens for subsequent analyses. Heat map and polynomial fitted curves intuitively presented the viral load changes in swabs collected from these 176 inpatients after admission (**Figures 2A, B**). The results showed that the viral copy number had peaked in most COVID-19 patients on admission, and the mean Cq on admission was 20.93. After treatment, the viral load rapidly decreased. The longest hospitalization duration was 29 days, the shortest was 6 days, and the median was 13 days (IQR 10.75–16 days). It is worth noting that after polynomial fitted curve calculation, the Cq on Day 8.54 of admission was 35, suggesting that the ***in vivo*** viral load decreased to a low level after 8.54 days of admission.

### 3.4 Infection characteristics of the virus in different sex and age groups

**Figure 3:**
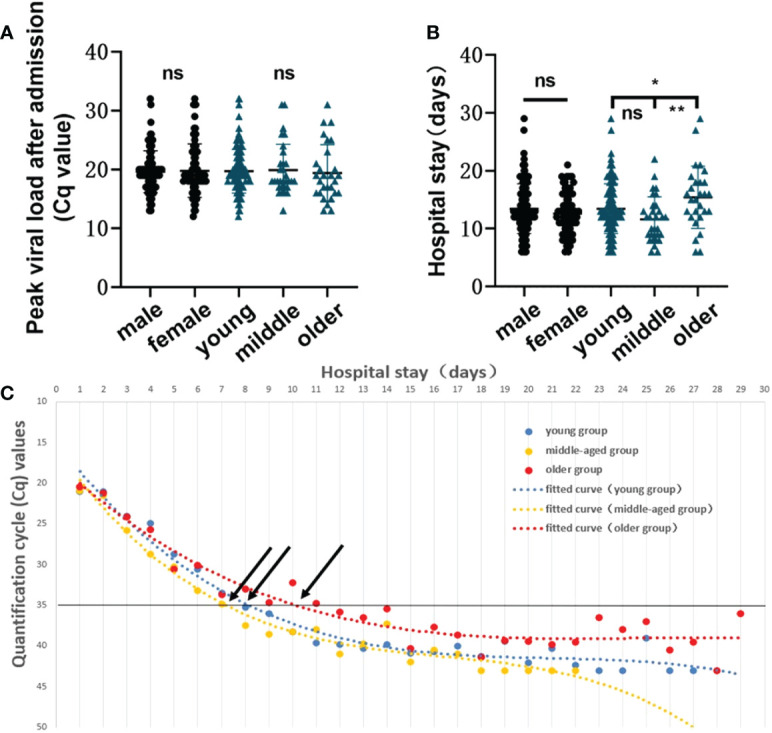
Infection characteristics of the virus in different sex and age groups. **(A)** Comparison of the peak viral load between sex groups and among age groups (young group, <40 years; middle-aged group, 40–60 years, and older group, >60 years). A t-test was used for comparisons between 2 groups, and ANOVA was used for comparisons among 3 groups. The same applies to the subsequent figures. **(B)** Comparison of length of hospitalization between sex groups and among age groups. ns, not significant, *p=0.0297, **p=0.0027. **(C)** Polynomial fitted curve analysis of mean Cq values among different age groups based on length of hospitalization. Black arrows show the intersection between the polynomial fitted curve and the Y-axis at 35.

To examine whether there are differences in COVID-19 infection predispositions and characteristics between sex groups and among age groups, we divided the study subjects by sex and age. T-test and ANOVA results showed that there were no significant differences in peak viral load (*p*=0.7700) or length of hospitalization (*p=*0.1451) between different sexes, and there was not a statistically significant difference in peak viral load among different age groups (*p*=0.9024). However, the mean hospitalization duration was longer in older than in young (*p*=0.0297) or middle-aged (*p* = 0.0027) patients (**Figures 3A, B**). Mean Cq polynomial curve fitting was conducted for the length of hospitalization for different age groups. The length of hospitalization for those with a Cq value ≈35 in the young, middle-aged, and older groups was 8.36, 7.40, and 10.14 days, respectively (**Figure 3C**). Thus, *in vivo* SARS-CoV-2 clearance is prolonged in older patients, and hospital stays are extended.

### 3.5 Relationship between viral load and disease severity

**Figure 4:**
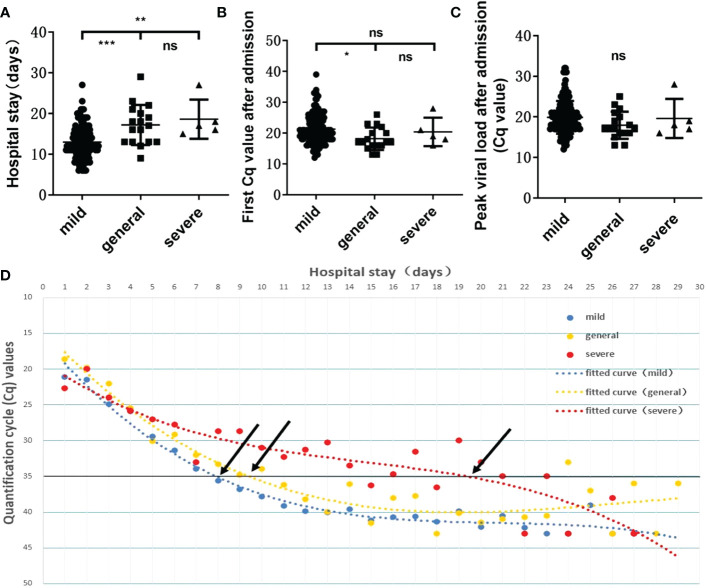
Relationship between viral load and disease severity. Relationship between the length of hospitalization **(A)** /first viral load after admission **(B)** /peak viral load during hospitalization **(C)** and disease severity. ns, not significant, *p=0.0250, **p=0.0069, ***p=0.0002. **(D)** Polynomial fitted curves showed the variation trends in viral clearance among disease severities. Black arrows show the intersection between the polynomial fitted curve and the Y-axis at 35.

It has been reported that the higher the *in vivo* viral load after SARS-CoV-2 infection is, the more severe the disease will be (11). Our statistical analysis revealed significant differences in hospitalization lengths between those with mild COVID-19 and those with general (*p*=0.0002) or severe disease (*p*=0.0069) (**Figure 4A**) and in Cq values on admission between those with mild and general disease (*p*=0.0250) (**Figure 4B**). No statistical significance was observed between the severe group and the mild/general group for single viral load on admission or peak viral load during hospitalization (**Figures 4B, C**). This may be due to the only 5 severe cases, different transit times, and irregular sampling times leading to such results. And for the multiple Cq values obtained during the patient**’**s hospitalization, we plotted polynomial fitted curves. The polynomial fitted curve demonstrated that *in vivo* virus shedding was faster in those with mild and general disease and slightly slower in those with severe disease. As shown in **Figure 4D**, a Cq value ≈35 was observed in patients with mild and general disease on days 8.09 and 9.37 of hospitalization, respectively, while a Cq value ≈35 was obtained in those with severe disease on day 19.60. This shows that we can further evaluate the patient**’**s condition based on their *in vivo* viral load on days 8–9 of admission to avoid overlooking disease severity. The above results indicated that disease severity is higher when *in vivo* virus clearance is slower.

### 3.6 Relationship between routine hematological indexes and disease severity

**Figure 5:**
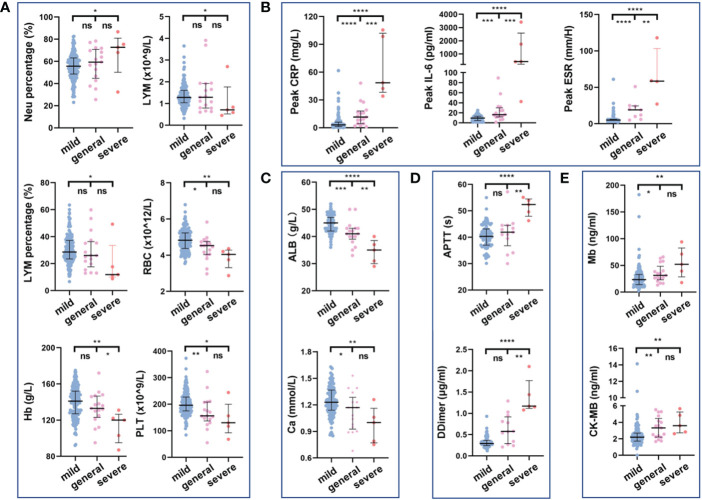
Relationship between routine hematology indexes and disease severity. Comparison of blood routine indexes **(A)**, inflammatory indexes **(B)**, biochemical indexes **(C)**, coagulation indexes **(D)**, and myocardial zymogram indices **(E)** among disease severities. ns, not significant, *p<0.05, **p<0.01, ***p<0.001, ****p<0.0001. The Kruskal–Wallis test was used for the statistical analysis.

We compared the routine hematological indexes of all COVID-19 patients with normal reference values (**Supplementary Table S2**). In addition, we further analyzed the routine hematological indexes of different types of patients (**Figure 5**; **Supplementary Table S3**). The first routine blood test results on admission showed that as disease severity increased, peripheral blood lymphocyte count and percentage decreased, which was particularly significant in those with severe disease compared with those with mild disease (*p*=0.0429 and *p*=0.0269, respectively); the corresponding neutrophil percentage was higher those with severe disease than in those with mild disease (**Figure 5A**). Red blood cell count, hemoglobin, albumin, and calcium ion values were lower in patients with more severe disease, suggesting a tendency toward anemia and abnormal hepatic function (**Figures 5A, C**). Coagulation disorders and elevated myocardial zymogram indices were more significant in severe COVID-19 patients (**Figures 5A, D, E**). In addition, CRP and IL-6 levels and the erythrocyte sedimentation rate during hospitalization were related to disease severity: the greater the disease severity was, the higher the inflammatory indexes were (**Figure 5B**). Abnormal increases or decreases in the above routine hematological indexes are related to patient condition and could aid clinicians in patient evaluations.

## 4 Discussion

This retrospective cohort study revealed the variation trends of viral load and routine hematological indexes in 176 inpatients infected with SARS-CoV-2. The clinical characteristics of the patients revealed that the symptoms of Omicron variant infection were mild, and the most common symptoms were cough, sore throat, and fever. Thus, the Omicron variant was less harmful to humans than other reported variants. The positive detection rate and viral load of respiratory tract samples were significantly higher than those in intestinal samples, and the positive detection rate of upper respiratory tract samples was higher than that of lower respiratory tract samples. A growing number of studies have shown that the Omicron variant affected mainly the upper respiratory tract and caused less severe pneumonia than other strains (12). Gupta et al. (13) from Cambridge University pointed out that the Omicron variant has a significantly reduced replication capacity in human pulmonary epithelial cells and intestinal epithelial cells compared to the wild-type strain and other previously concerned variants. Entry of SARS-CoV-2 *via* cell surface fusion requires activation of the spike protein by both host angiotensin converting enzyme 2 (ACE2) and transmembrane protease serine 2 (TMPRSS2) (14). Therefore, the team confirmed that the difference in replication ability of different variants of SARS-CoV-2 in these two cell lines may be related to the fact that the spike protein of Omicron carries multiple mutations located at the protease cleavage site, causing a reduced ability to utilize TMPRSS2, affecting the viral invasion ability and thus leading to its diminished replication ability. In another study, mice infected with omicron had 10- to 100-fold lower viral loads in the lungs than other variants (15).The diminishing pathogenicity and increasingly mild symptoms of the omicron variant may also be associated with improved vaccination rates.The body stimulates the immune system to fight the virus because of vaccines or infections, thus pressuring the SARS-CoV-2 genetic mutations to try to get rid of the human immune system, and these genetic mutations also simultaneously reduce virus replication in the lungs. With this development, vaccination including additional booster injections may further direct viral mutation and weakening. These results also showed that upper respiratory tract samples are particularly important for detecting the Omicron variant.

In this study, we found that the viral load of SARS-CoV-2-positive patients peaked on admission (mean Cq on admission: 20.93) and gradually decreased with treatment. Previous researchers have reported that COVID-19 is similar to severe acute respiratory syndrome (SARS) in that the higher the viral load is, the more severe the infection will be (16–18). In this study, we only observed significant differences in viral load on admission between the mild and general groups, and did not observe statistically significant differences between the severe group and the mild/general group for single viral load on admission or peak viral load during hospitalization. Because the symptoms triggered by this Omicron variant subtype BA.2.2.1 tended to be mild, we collected only five severe cases in this study. Thus, the small size number might be one of the reasons why there was no statistical significance between the severe group and the mild/general group. In addition, both the transit time from symptom onset to hospitalization and the sampling time may result in different viral loads at admission as well as peak viral loads. These factors may have influenced our interpretation of the results. What is of interest is that we found that *in vivo* virus clearance was correlated with the patient**’**s age and condition by analyzing viral shedding kinetics. Our results showed that the time needed to reach a Cq value of 35 was longer in older patients and patients with more severe disease. Hence, the length of hospitalization was increased. Therefore, additional attention should be paid to prevention in older adults with underlying diseases and poorer immunity during the COVID-19 epidemic.

In this study, viral load was determined by RT-qPCR, using the parameter Cq value. Cq value is the number of cycles that the fluorescent signal in each reaction tube undergoes to reach a set detection threshold and does not reflect the true number of viral copies in the sample. At present, digital PCR technique, which allows absolute quantification of viral load, is a good choice. However, due to the complicated steps, low detection flux, and long detection time, digital PCR has not been widely used in clinical. Studies have shown that the Cq value of each template is linearly related to the logarithm of the starting copy number of that template, with the higher the starting copy number, the lower the Cq value, and vice versa (19). Cq values can be affected by a number of factors such as primer probe design, fluorescent groups, amplification efficiency, reaction system, instrument platform, software optimization, etc., resulting in Cq values that cannot be compared as a constant quantity. Individual Cq values in different laboratories can correspond to different viral load. Therefore, we should take into account the possible inaccuracy and incomparability between laboratories when using Cq values as a substitute for viral load to analyze viral shedding pattern and infectivity.

In addition, to provide laboratory data related to disease severity in clinical practice, we investigated the routine hematological indexes of these patients. The first tests on admission revealed that patients with decreased lymphocyte counts and percentages, anemia, coagulation disorders, abnormally elevated myocardial zymogram indices, and decreased albumin and calcium levels might have more severe disease. In addition, abnormal elevation of inflammatory indexes, such as CRP and IL-6, and the erythrocyte sedimentation rate also suggest worsening disease; thus, attention should be paid to these indexes in clinical practice.

## Data availability statement

The original contributions presented in the study are included in the article/**Supplementary Material**, further inquiries can be directed to the corresponding authors.

## Ethics statement

The studies involving human participants were reviewed and approved by the medical Ethics Committee of Changhai Hospital. The patients/participants provided their written informed consent to participate in this study. Written informed consent was obtained from the individual(s) for the publication of any potentially identifiable images or data included in this article.

## Author contributions

SL, YJ and XZ designed the study. XZ, XH and TS performed the data collection, data analysis and prepared the manuscript. XJ, ZT, MX, and JK performed the data collection and analysis. AX and YC performed the Clinical consultation. BG provided the information data. SL and YJ revised and approved the manuscript. All authors contributed to the article and approved the submitted version.
