# Supplementary material for: Chronological changes of viral shedding in adult inpatients with Omicron infection in Shanghai, China
Source: Front Immunol. 2023 Feb 1;14:1090498. doi: 10.3389/fimmu.2023.1090498 (PMC9929285; doi:10.3389/fimmu.2023.1090498)

Supplementary Material

Supplementary Table

**Supplemental Table S1.** Comparison of COVID-19 nucleic acid test results between matched specimens

| Type of specimen | Nasopharyngeal swab and oropharyngeal swab | Induced sputum | Anal swab |
| --- | --- | --- | --- |
| Total number of specimens | 360 | 360 | 360 |
| Number of positive specimens | 282 | 248 | 41 |
| Positivity rate (%) | 78 | 69 | 11 |

**
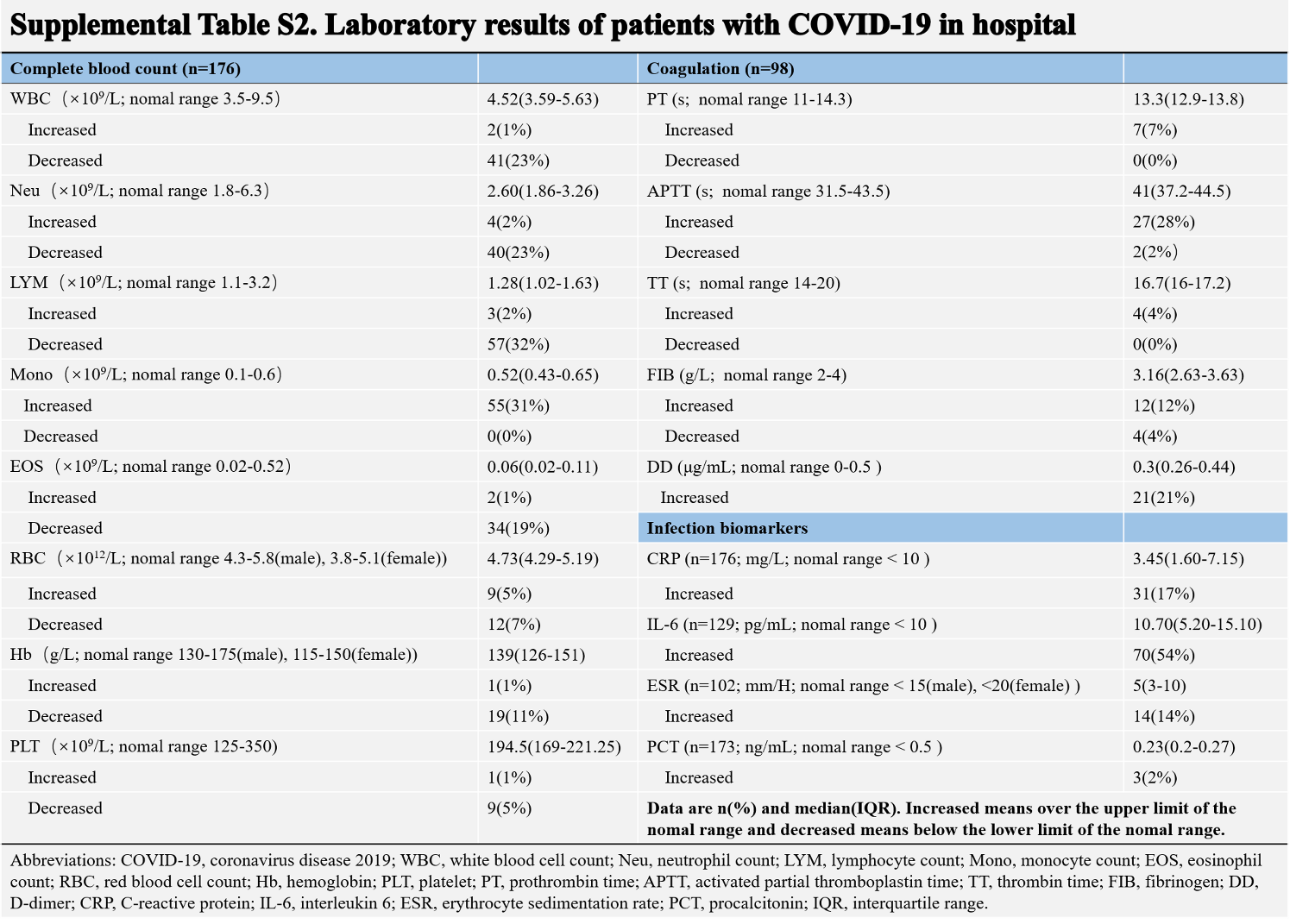
**

**Supplemental Table S3.** Laboratory results of patients with COVID-19 in hospital

| The first time after hospitalization | Mild（n=154）  Median(IQR) | General（n=17）Median(IQR) | Severe（n=5）  Median(IQR) | p值  (Kruskal-Wallis test) |
| --- | --- | --- | --- | --- |
| Complete blood count |  |  |  |  |
| WBC,×109/L | 4.46(3.55-5.54) | 5.19(3.77-6.85) | 5.21(4.68-5.48) | 0.2809 |
| Neu,×109/L | 2.565(1.85-3.21) | 2.79(1.95-4.54) | 3.18(2.75-3.93) | 0.2262 |
| Mono,×109/L | 0.53(0.43-0.66) | 0.50(0.42-0.60) | 0.58(0.51-0.66) | 0.6675 |
| Mono percentage,% | 12.05(9.63-14.68) | 10.30(8.60-12.50) | 12.70(12.40-13.50) | 0.2436 |
| EOS,×109/L | 0.05(0.02-0.11) | 0.08(0.02-0.19) | 0.03(0-0.06) | 0.1545 |
| EOS percentage,% | 1.20(0.60-2.58) | 1.70(0.50-2.80) | 0.60(0-1.50) | 0.2144 |
| BASO,×109/L | 0.015(0.01-0.02) | 0.01(0.01-0.02) | 0.01(0.01-0.02) | 0.4878 |
| BASO percentage,% | 0.30(0.20-0.50) | 0.30(0.20-0.40) | 0.30(0.10-0.40) | 0.1736 |
| Coagulation |  |  |  |  |
| PT,s | 13.20(13-13.73) | 13.40(12.30-14.00) | 13.7(13.40-14.20) | 0.4396 |
| PTINR | 1(0.98-1.05) | 1.02(0.91-1.08) | 1.05(1.02-1.10) | 0.4396 |
| TT,s | 16.50(15.98-17.1) | 17(16.1-19.3) | 18.4(16.80-19) | 0.0718 |
| FIB,g/L | 3.06(2.605-3.5225) | 3.29(2.90-3.96) | 5.17(3.25-5.23) | 0.1541 |
| Electrolytes |  |  |  |  |
| K,mmol/L | 104(102-106) | 105(102-108) | 104(103-104) | 0.7983 |
| Na,mmol/L | 3.90(3.70-4.20) | 3.70(3.50-40) | 3.80(3.10-4.10) | 0.1405 |
| Cl,mmol/L | 4.10(3.90-4.60) | 4.60(4.20-5.20) | 4.60(4.30-6.20) | 0.0163 |
| IP,mmol/L | 0.87(0.82-0.93) | 0.84(0.79-0.91) | 0.81(0.75-0.83) | 0.1740 |
| Liver function |  |  |  |  |
| ALT,U/L | 15(11.25-21) | 18(13-46) | 11(11-19) | 0.2552 |
| AST,U/L | 18(15-22) | 23(19-34) | 20(14-20) | 0.0194 |
| ALP, U/L | 52(44-60) | 59(51-77) | 52(45-96) | 0.0523 |
| GGT, U/L | 17(13-22) | 24(14-50) | 15(13-71) | 0.0266 |
| LDH, U/L | 173.5(156.25-194) | 195(176-215) | 195(183-203) | 0.0316 |
| Glu,mmol/L | 73.5(58-87.75) | 79(59-89) | 107(95-118) | 0.0184 |
| Myocardial enzyme |  |  |  |  |
| cTnl, ng/ml | 0.05(0.05-0.12) | 0.05(0.05-0.11) | 0.05(0.05-0.05) | 0.6992 |
| Peak value after hospitalization |  |  |  |  |
| PCT, ng/ml | 0.22(0.19-0.26) | 0.26(0.23-0.31) | 0.28(0.27-0.28) | 0.0015 |

Abbreviations: COVID-19, coronavirus disease 2019; IQR, interquartile range; WBC, white blood cell count; Neu, neutrophil count; Mono, monocyte count; EOS, eosinophil count; BASO, basophil count; PT, prothrombin time; PTINR, prothrombin time international normalization; TT, thrombin time; FIB, fibrinogen; K, Kalium; Na: natrium; Cl: chlorine; IP, inorganic phosphate; ALT, alanine transaminase; AST, aspartate transaminase; ALP, alkaline phosphatase; GGT, γ-glutamyl transferase; LDH, lactate dehydrogenase; Glu, glucose; cTnI, Cardiac Troponin I; PCT, procalcitonin.

Supplementary Figure

**Supplemental Figure S1.** Macrogenome sequencing analysis of two blindly selected samples
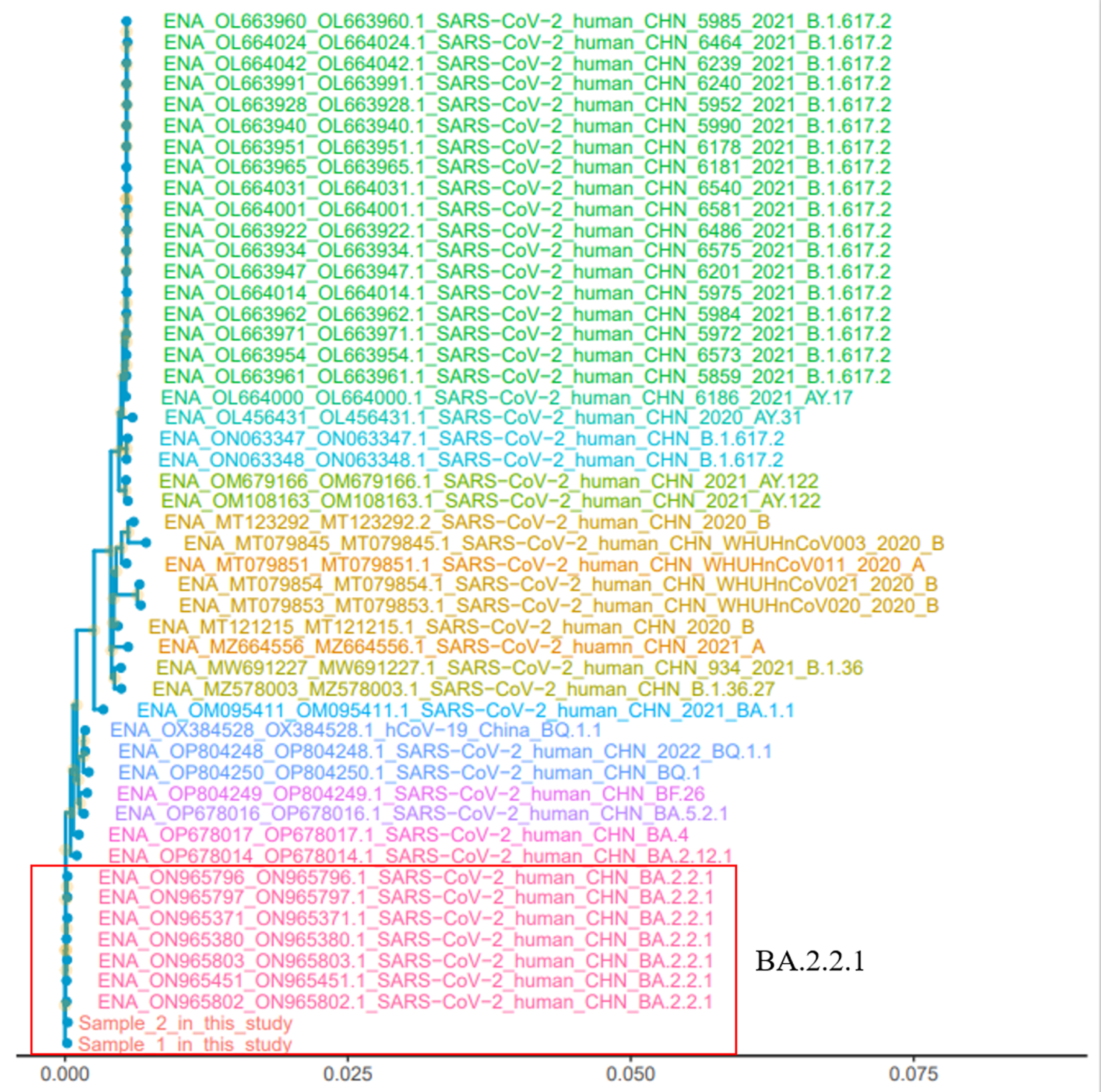


**Supplemental Figure S2.** Course of Omicron inpatients with combined autoimmune diseases

**
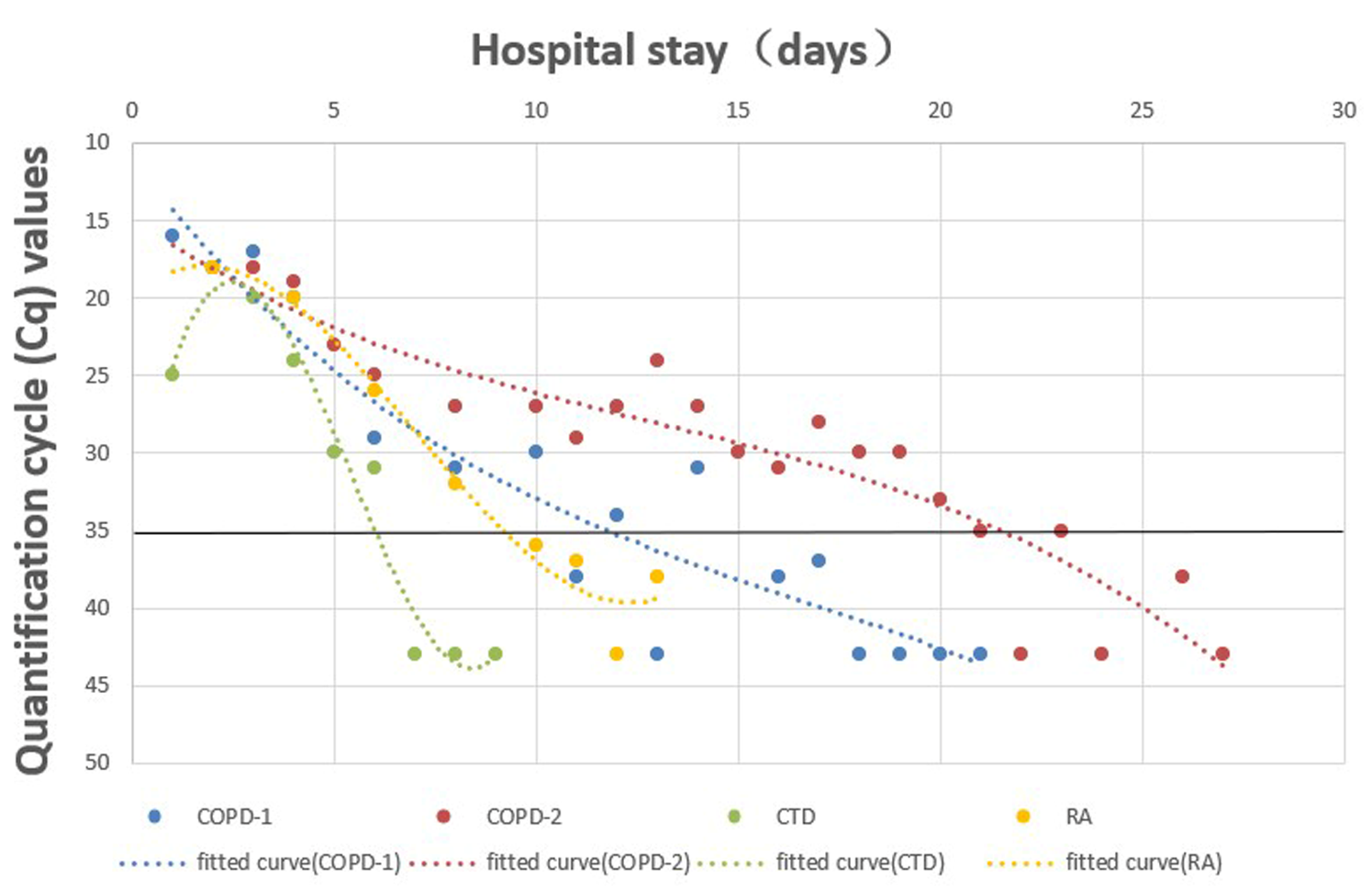
**

**Supplemental Figure S3.** Effect of molnupiravir on the course of the disease


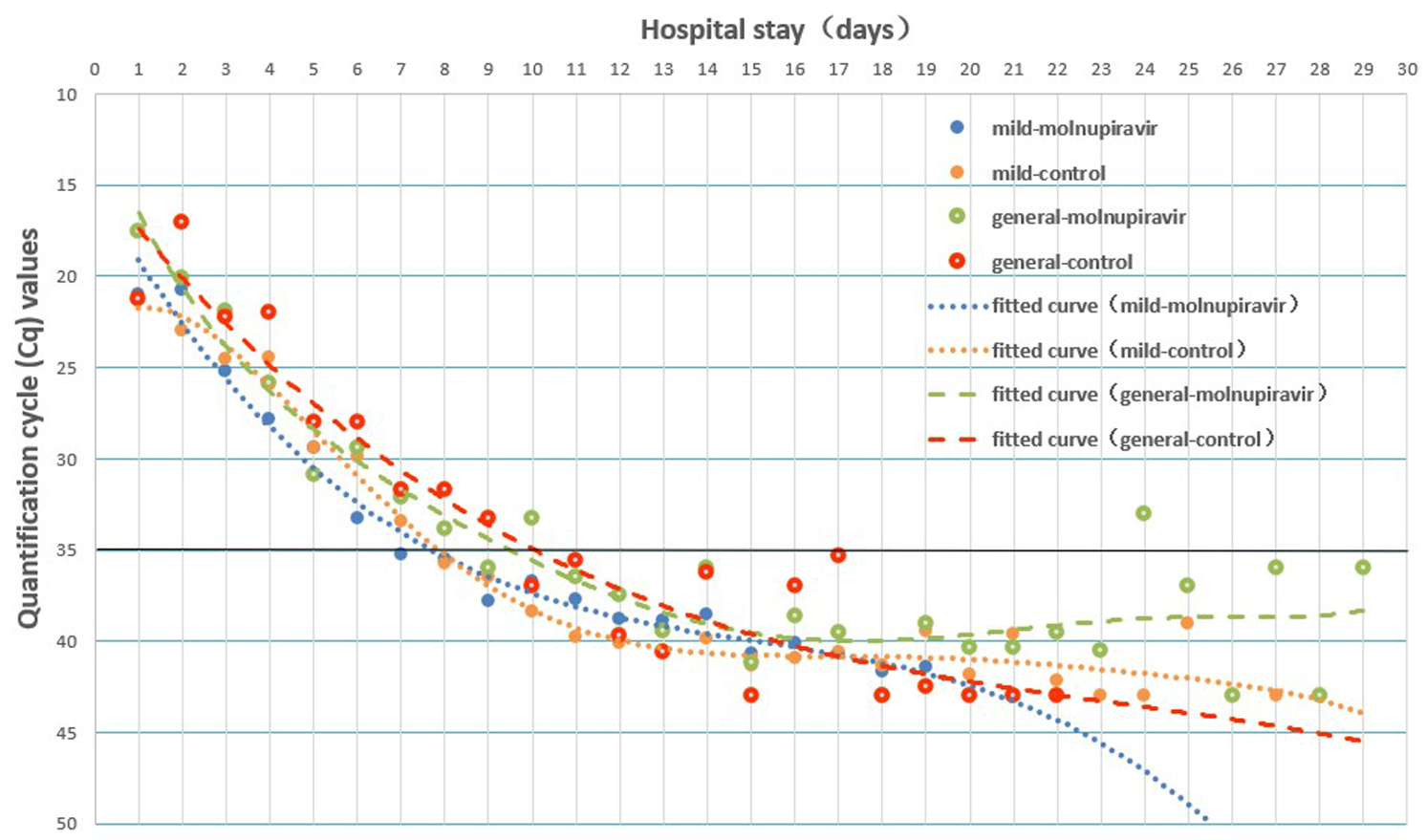

Supplement: Supplementary file 1 [file DataSheet_1.docx]
